# Computational Fracture Evolution Analysis of Steel-Fiber-Reinforced Concrete Using Concrete Continuous Damage and Fiber Progressive Models

**DOI:** 10.3390/ma16165635

**Published:** 2023-08-15

**Authors:** Iwona Pokorska, Mariusz Poński, Wojciech Kubissa, Tomasz Libura, Adam Brodecki, Zbigniew Kowalewski

**Affiliations:** 1 Institute of Fundamental Technological Research, Polish Academy of Sciences, 02-106 Warszawa, Poland; mponski@ippt.pan.pl (M.P.); tlibura@ippt.pan.pl (T.L.); abrodec@ippt.pan.pl (A.B.); zkowalew@ippt.pan.pl (Z.K.); 2 Faculty of Civil Engineering, Cza̧stochowa University of Technology, 42-218 Czȩstochowa, Poland; 3 Faculty of Civil Engineering, Mechanics and Petrochemistry, Warsaw University of Technology, 09-400 Płock, Poland; wojciech.kubissa@pw.edu.pl

**Keywords:** concrete, finite element method (FEM) simulations, steel-fiber-reinforced concrete (SFRC), crack mouth opening displacement (CMOD), steel fibers

## Abstract

The process of concrete cracking is a common problem because the first micro-cracks due to the loss of moisture may appear even before the concrete is loaded. The application of fracture mechanics allows for a better understanding of this problem. Steel-fiber-reinforced concrete (SFRC) samples with a notch were subjected to a three-point bending test, and the results for crack energy were used to analyze the concrete’s material properties. In this paper, an experimental and numerical analysis of SFRC with rapid changes in the force (F) crack mouth opening displacement (CMOD) curve (F-CMOD) is presented. In order to obtain the relevant F-CMOD diagrams, three-point bending tests were carried out with non-standard samples with a thickness equal to one-third of the width of standard samples. For analysis purposes, crimped steel fibers were adopted. A probabilistic analysis of the most important parameters describing the material in question, such as peak strength, post-cracking strength, crack mouth opening displacement (CMOD), fracture energy, and the post-cracking deformation modulus, was conducted. The tests and the analysis of their results show that the quasi-static numerical method can be applied to obtain suitable results. However, significant dynamic effects during experiments that influence the F-CMOD curves are hard to reflect well in numerical calculations.

## 1. Introduction

Concrete is the most widely used construction material in the building industry due to its versatility, durability, and feasible application. In order to meet the modern requirements of increasingly complex engineering structures, concrete reinforcements are applied to ensure the desired strength and adequate safety. The porous, heterogeneous structure of concrete with internal imperfections needs to be strengthened to improve its inherently low tensile strength, poor fracture toughness, brittleness, low deformation capacity, and low energy absorption [1,2,3].

In order to strengthen and improve the mechanical properties of concrete, various types of fibers can be applied to obtain a fiber-reinforced composite (FRC). Carbon or stainless steel fibers are used in FRCs [4]. However, they can also be made of polymers, glass, and natural or recycled materials [5].

Glass, aramid, polypropylene, and basalt fiber additives [6,7,8,9] can inhibit the growth and propagation of cracks and, thus, affect the durability of concrete composites. The application of hybrid fibers of various types at variable scales and lengths [10] can increase the strength properties and provide positive synergy effects [11,12,13].

The most commonly used fibers in concrete composites are steel fibers (i.e., steel-fiber-reinforced concrete, or SFRC). The structural role of this type of fiber is to prevent or delay the propagation of cracks. Fibers can be treated as creating a bridging effect that also allows a homogeneous stress distribution in the concrete matrix [12,14,15,16] to ensure the residual strength after fracture [17]. Such fibers also increase the tensile flexural strength, fatigue strength, and impact strength of concretes (FRCs) [18,19,20,21].

The strength of steel fibers, their geometry (final shape, cross-section, diameter), the length of their embedding, their orientation, and their anchoring in concrete determine the strength of the bonds between the fibers and concrete, which plays an essential role in their tearing out due to the behavior of the composite matrix [22,23]. Steel fibers can differ in length and shape and include straight, hooked, wavy, spiral, flattened, and twisted [24] fibers. Ha Vinh Ho et al. [24] conducted research on different wave sizes in crimped fibers made of cold-drawn steel. Corrugated fiber has the most desirable shape, which can significantly increase the pull-out resistance. The application of a sufficient number of steel fibers in concrete can affect the ULS, and, as a consequence, the fibers can partially or entirely replace the conventional reinforcement.

If the orientation of steel fibers in the structural elements is consistent with the direction of the main tensile stress, their ductility will be increased. Longbang Qing et al. [25] found during tensile tests that the uniform distribution of steel fibers in the concrete mix achieved due to the usage of a magnetic field led to an increase in the tensile strength, post-break energy dissipation, and impact strength. Destructive tests of the reinforced concrete composite enabled the assessment of the effectiveness of the steel fibers in crack propagation prevention, on the one hand, and a better understanding of the mechanism of the fibers’ tensile process, on the other. Steel fibers do not provide the tensile strength of SFRC. The strength of the bonds between the steel fibers and the concrete plays an important role in such cases. Two types of bonds can be distinguished between the cement matrix and fibers, i.e., mechanical and physicochemical. The first is responsible for the anchoring of braided fibers in cement [26], whereas the second acts through friction and adhesion, depending on the fiber’s surface and the properties of the interfacial zone. When straight fibers are used in SFRC, only physicochemical bonds occur, and they define the tensile strength of straight fibers. In the case of fibers with variable geometry, mechanical bonds occur. The contact surface and the strength of the concrete against pressure determine the mechanical interlock [27].

In the literature related to the analysis of fiber-reinforced-concrete cracking, one can find various combinations of constitutive models adapted for concrete and for fiber material. An extensive review of both continuum and discrete mechanical concrete models, including crack simulation, was presented by Bolander et al. [28]. The following is an overview of the most common constitutive concrete models. The Coulomb–Mohr (C-M) model is often used in engineering practice to determine the strength of concrete and other brittle bodies [29,30]. This is due to its high accuracy and the small number of parameters. Soil mechanics and, partly, rock mechanics are based on this model. It requires the definition of the following parameters: Young’s modulus, angle of internal friction, Poisson’s ratio, cohesion, and dilation angle. The yield surface determined with the C-M model is represented as an irregular pyramid with a base consisting of an equilateral hexagon with different angles in the three-dimensional stress space defined by three limit functions. It is independent of the hydrostatic pressure.

The Menétrey–Willam (M-W) model [31,32] is also used to describe the mechanical response of concrete under multiaxial compression conditions by means of the yield surface concept. In this approach, the yield surface changes itself while maintaining the concentricity of the hydrostatic axis. The model assumes isotropic hardening. The M-W model takes into account many effects and properties of the mechanical behavior of concrete, such as softening, nonlinear hardening, various tensile and compressive strength, and expansion joints.

The Drucker–Prager constitutive model [33,34] (D-P), as a generalization of the Coulomb–Mohr criterion, was initially used to model geological materials such as soils, rocks, clays, and other materials such as concretes, polymers, and foams. It is a three-dimensional model described by three stress tensor invariants. The yield criterion of the D-P model in the form of octagonal normal and shear stress components is based on the assumption that there is a linear relationship between octahedral stress components through material constants. It allows us to determine whether the material was plastically deformed or damaged. Since the traditional D-P models did not take into account the softening after cracking or crushing, they were modified accordingly. In addition, the D-P criterion with isotropic hardening and softening does not describe the actual behavior of the concrete due to the linear nature between the average stress and the stress intensity and due to the independence from the third stress tensor invariant. Therefore, further model proposals were developed to reflect the required plastic deformation conditions.

Willam and Warnke [35,36] (W-W) combined the Coulomb–Mohr and Drucker–Prager criteria. They formulated the third and fifth parameters of the W-W plasticity criteria for concrete, in which the boundary surfaces in the area of medium compressive hydrostatic stresses consist of parabolic meridians, while in the area of high values the surfaces are linear. However, the boundary surface is described by three tangent segments of ellipses in a deviatoric space, and the surface is assumed to be non-rotating. In turn, the four-parameter failure criterion was proposed by Ottosen [37] assuming a non-circular section on the deviatoric plane. It assumes the surfaces of the failure envelope open towards the hydrostatic pressure in the stress space, which proves that the material should not undergo plastic deformation even under high hydrostatic pressure.

The contact density model [38,39] was formulated on the basis of continuous damage mechanics (CDM). The crack surface is presented as a set of potential, infinitely small contact planes between which there is contact stress acting perpendicularly to their surface. It has a rigid-plastic or elastic-plastic nature. The planes are defined by the shape density function. The model assumes a physicomechanical relationship dependent on the aggregate interlock. In contrast, in the concrete damage plasticity (CDP) model [40,41], an evolution of the yield surface combines the plastic deformation with damage mechanics, i.e., compression crushing with tension fracture, based on the Lubliner yield surface. This model is used to reflect the mechanical response to damage to the cement mortar, concrete, or brick.

The parabolic total strain crack model [42,43] represents another group of continuous mechanics approaches based on a fuzzy attempt to fracture energy. It is used in the analysis of concrete structures, assuming concrete as a non-linear, isotropic, homogeneous material. The constitutive equations describing the model are based on the stress–strain relation. The calculations of the model are more straightforward due to the generation of only normal stresses on the crack surface. The direction of the crack remains parallel to the principal strain. The cracking criterion is based on softening, which depends on the crack opening and occurs when the tensile strength of the concrete is exceeded.

Another type of constitutive model is a model of microplates [44,45]. The basic idea of such constitutive equation sets is to replace the relationship between strain and stress tensor invariants with relationships between strain and stress vectors acting on differently oriented microplates that make up a spherical surface. Macroscopic stresses are calculated by integrating deviator, volumetric, and shear microscopic stress components in the microplates. The applied vector approach allows us to determine the oriented damage types: slip, tensile cracking, compression splitting, friction, and fiber reinforcement orientations. A detailed description of its modifications is given by Bazant et al. [46].

The actual concrete fracture specific energy $G_{f}$ is considered a useful material parameter in the analysis of concrete structures [47,48]. The test method is not clearly defined, and even the size of the samples used by different researchers is not the same. The article [49] presents the results of tests and analysis in the case of using samples with different proportions of length, width, and thickness subjected to three-point bending. The authors are still looking for new dependencies of the shear stress on deformation or crack opening. New formulas, sample shapes, and test methods are introduced. An example of using a new relationship to determine the shear strength of concrete with recycled aggregate and its confirmation in experimental research is presented in the article [50]. The authors of [51] presented tests of eccentrically loaded notched samples subjected to a long-term (about a month) increase in load over time, during which the crack opening was measured. The effect of scale was analyzed by examining samples with different dimensions.

Another approach to the study of the problem of cracking fiber-reinforced concrete composites was proposed by the authors of the article [52]. Bearing in mind the heterogeneity of the concrete composite, they used a model for mesoscale brittle materials, i.e., the lattice discrete particle model (LDPM) [53], extending it to include fibers as discrete dispersed particles in a cement matrix (LDPM-F). They carried out verifications of the basis for the use of the model, employing Schauffert and Cusatis calibrations and validations of the model using numerical summations of three-point bending for concrete cylindrical samples with different numbers of steel and synthetic fibers. This allowed them to obtain a response to the variables and evolving mechanical properties of the fibers over time, and to determine their strength and ductility, and thus accurately assess the fracture resistance of the concrete composite. Another approach to the cracking properties of concrete composites with steel fibers was proposed by Montero-Chacón et al. [54], who adopted the lattice–particle model for the SFRC strength analysis.

The authors improved the model by adopting explicit modeling of the fibers using interference elements between them and the cement matrix. In addition to the mechanical properties, the model also takes into account the geometric properties of the SFRC. The model adopted in this way was verified by means of uniaxial tensile and compression tests and analytical calculations. The results show that an increase in the fiber misorientation angle leads to a decrease in plasticity, while an increase in volume fractions ($V_{f}$) leads to an increase. These parameters were used to determine the cracking properties of the SFRC. The adopted model was combined with the homogenization method. The representative element analysis (RVE) was performed. The applied multiscale modeling was successfully tested with three-point bending.

Using cracking energy parameters, the authors of the article presented another approach based on identifying the mechanical properties of a concrete composite reinforced with steel fibers. A probabilistic analysis of the most important parameters describing the tested material was used. The constitutive Menétrey–Willam model was used for the analysis. Experimental studies were carried out on a larger number of samples (30 pcs) to solve the problem. A non-standard sample thickness of 1/3 of the width of standard samples was used, as well as an original approach to numerical calculations.

## 2. Materials and Methods

### 2.1. Materials

Portland cement CEM I 42.5R produced by the Górażdże Cement Plant located in Chorula, Poland.according to the PN-EN 197 Standard was used. The basic physical and chemical properties presented by the cement manufacturer are shown in Table 1.

The concrete mixture contained 380 $\mathrm{kg}/m^{3}$ of cement with a 0.44 w/c ratio. A fraction of river sand of 0–2 mm and a natural gravel fraction of 2–8 mm were applied. Aggregates were stored in laboratory air-dry conditions. The sand point was established to be SP = 37%, and 12% sand and 88% gravel were used, which allowed the aggregate grading curves to fit between the boundary curves. Superplasticizer Atlas Duruflow PE-220 and VMA admixture Atlas Duruflow VM-500were used according to PN-EN 934-2. Regular tap water was applied as the mixing factor. Steel fibers made of low-carbon steel C4D1 produced by Arcelor were used; these had a wavy shape, a diameter of 0.8 mm, and a length of 50 mm.
**Proportions of concrete mixtures (kg/m^3^)**
**Material****Mixture ID****F1/F2**cement CEM I 42.5R380natural sand 0–2 mm    220gravel 2–8 mm1611steel fibers25water167SP PE-220 % m.c.1VMA VM-500 % m.c.0.2

**Figure 1:**
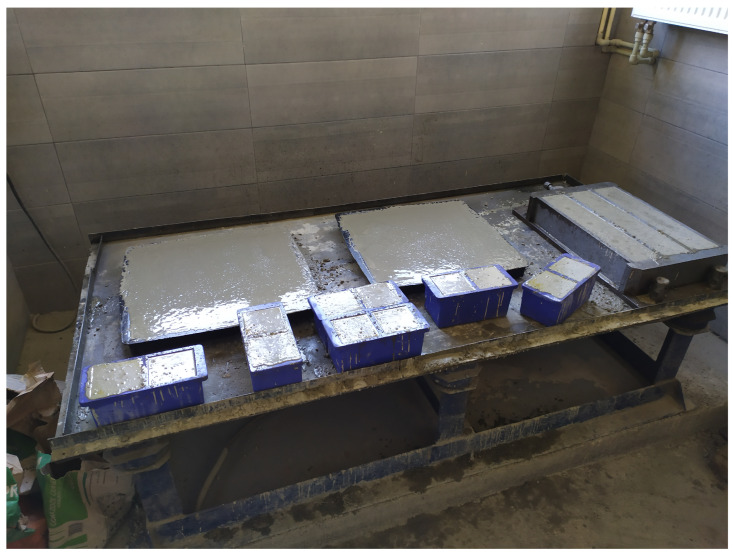
Sample preparation for mechanical properties tests (description in text).

**Figure 2:**
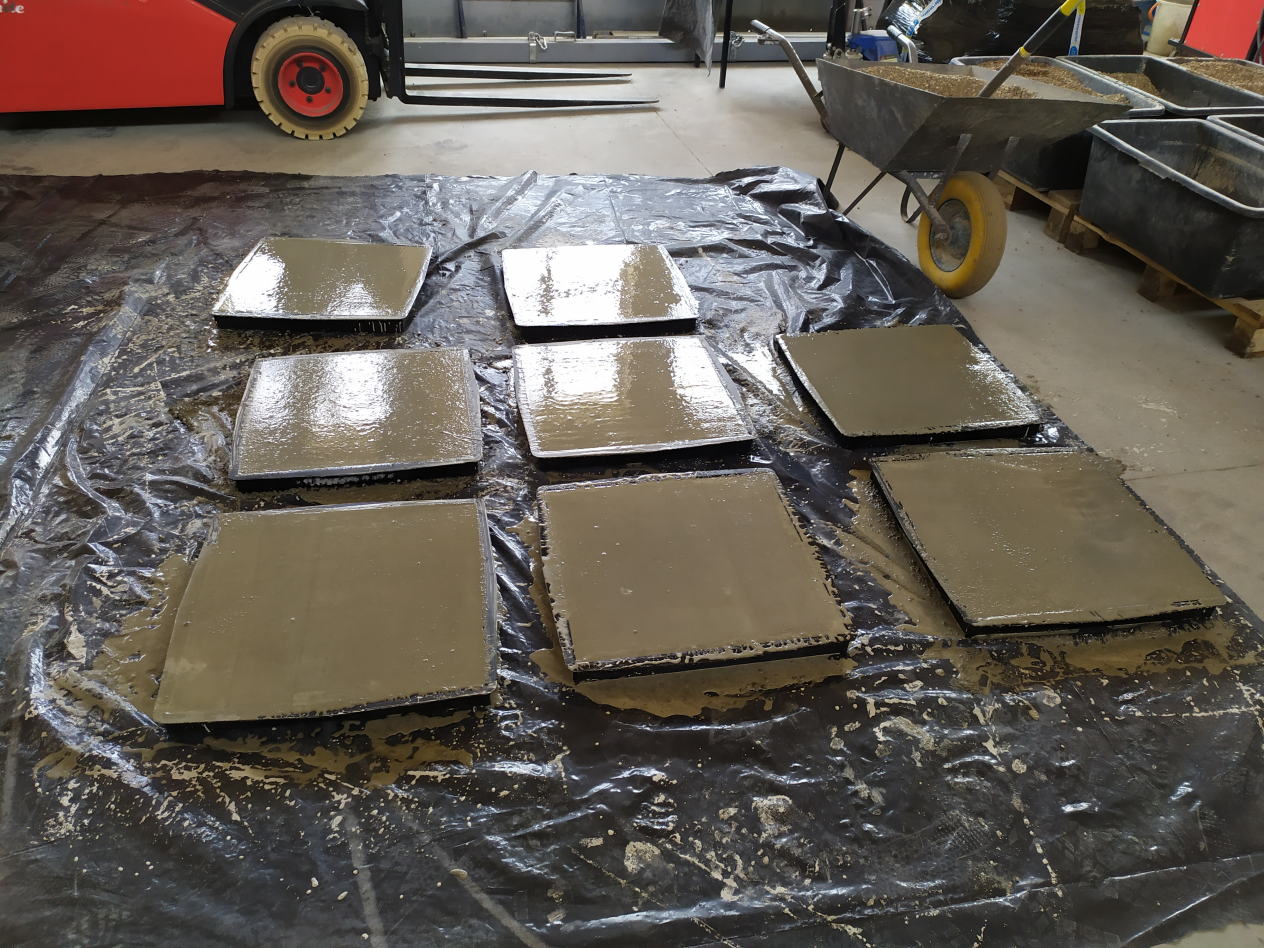
Sample preparation for major experiment (description in text).

Concrete mixtures were prepared in two batches, F1 and F2. Mix proportions are presented in Table 2. The consistency of fresh concrete was measured via table flow test in accordance with PN-EN 12350-5. Specimens in the form of 100 mm cubes (Figure 1) for the compressive strength testing were prepared and cured according to PN-EN 12390-2. They were cast in plastic molds and compacted by double vibration (half and full) on a vibrating table. After one day, they were stripped, and then they were water-cured in the laboratory for 28 days. Specimens were also made for compressive strength testing (100 mm cube) and for flexural tensile strength testing (500 × 100 × 100 mm beams, Figure 1). A total of 30 samples for each test were made in this way, which, after removal from the molds, were stored together with notched beams under air-dry laboratory conditions t = 20 °C ± 2 °C and RH = 50% ± 10% and tested after 134 days. The slabs from which the notched beams were cut were made in horizontally placed molds with dimensions of 500 × 500 × 50 mm, as shown in Figure 2. Using the experience of [56,57], it was decided to form the specimens horizontally, which allows better compaction and homogeneity of the mixture than the application of vertical forms. The specimens were molded and compacted in two layers. In order to minimize the segregation of the mix and separation of water on the top surface, it was decided to use an F2 mix consistency that allows proper compaction of straight-shaped samples, and VMA was admixed. This method of making the samples ensured an even distribution of steel fibers throughout the sample volume. After about 20 days of molding, 500 × 200 × 50 mm specimens were cut from the plates using a lab saw, and a 10 mm high, 4 mm wide notch was cut in the middle of the longest wall. According to [46], the correlation length for concrete is between 45 and 75 mm. In connection with the above, the assumed width was taken as 50 mm.

### 2.2. Mechanical Properties

**Figure 3:**
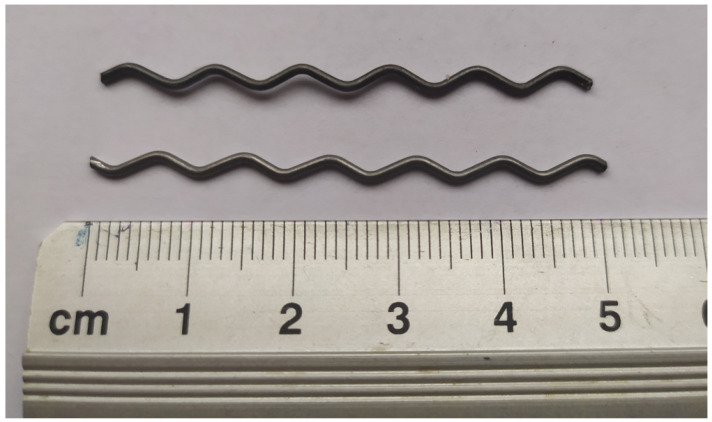
Steel fibers used in analysis.

The compressive strength tests were conducted on 100 mm cube specimens after 28 and 134 days of hardening. The tests were carried out following PN-EN 12390-3 using a ToniTechnik instrument of 3000 kN compression force capacity. The flexural strength test was conducted on the beam with dimensions of 500 × 100 × 100 mm after 134 days of hardening. The test was carried out in accordance with PN-EN 12390-5 using a Matest two-frame instrument of 300 kN compression force capacity. The loading rate was maintained at 0.5 MPa/s for the compressive strength test and 0.05 MPa/s for the flexural strength test, as shown in Figure 3.

The mechanical parameters of the compressive tests are summarized in Table 2. The table also presents the average values of all parameters captured. The difference between the compressive strength of the F1 and F2 concrete after 134 days is insignificant. Also, no essential differences were found in the tensile strength for concrete F1 and F2. The average values of parameters for the two series, F1 and F2, were taken for further calculations. Table 2 presents the average values and their standard deviations.

### 2.3. Experimental Beam Tests

**Figure 4:**
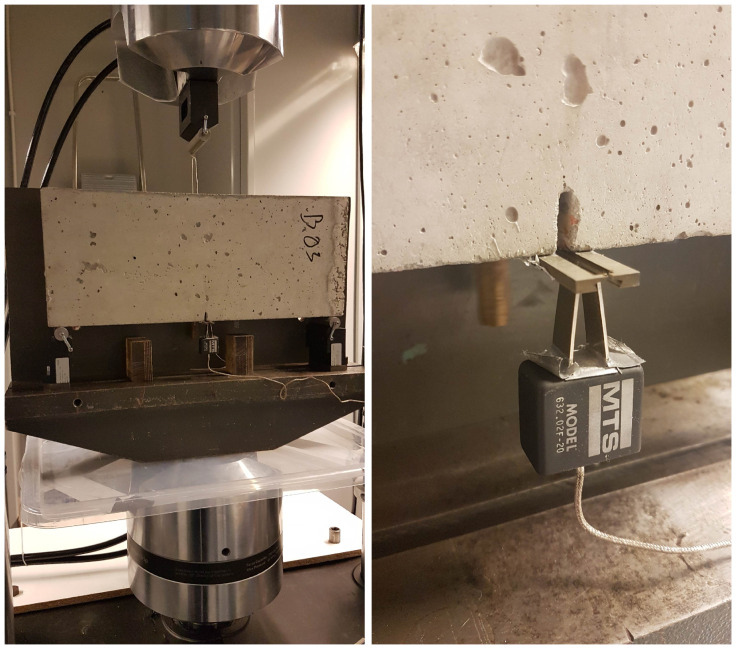
The sample set on the measuring stand (**left panel**); view of the measuring part of the sample with the extensometer attached (**right panel**).

**Figure 5:**
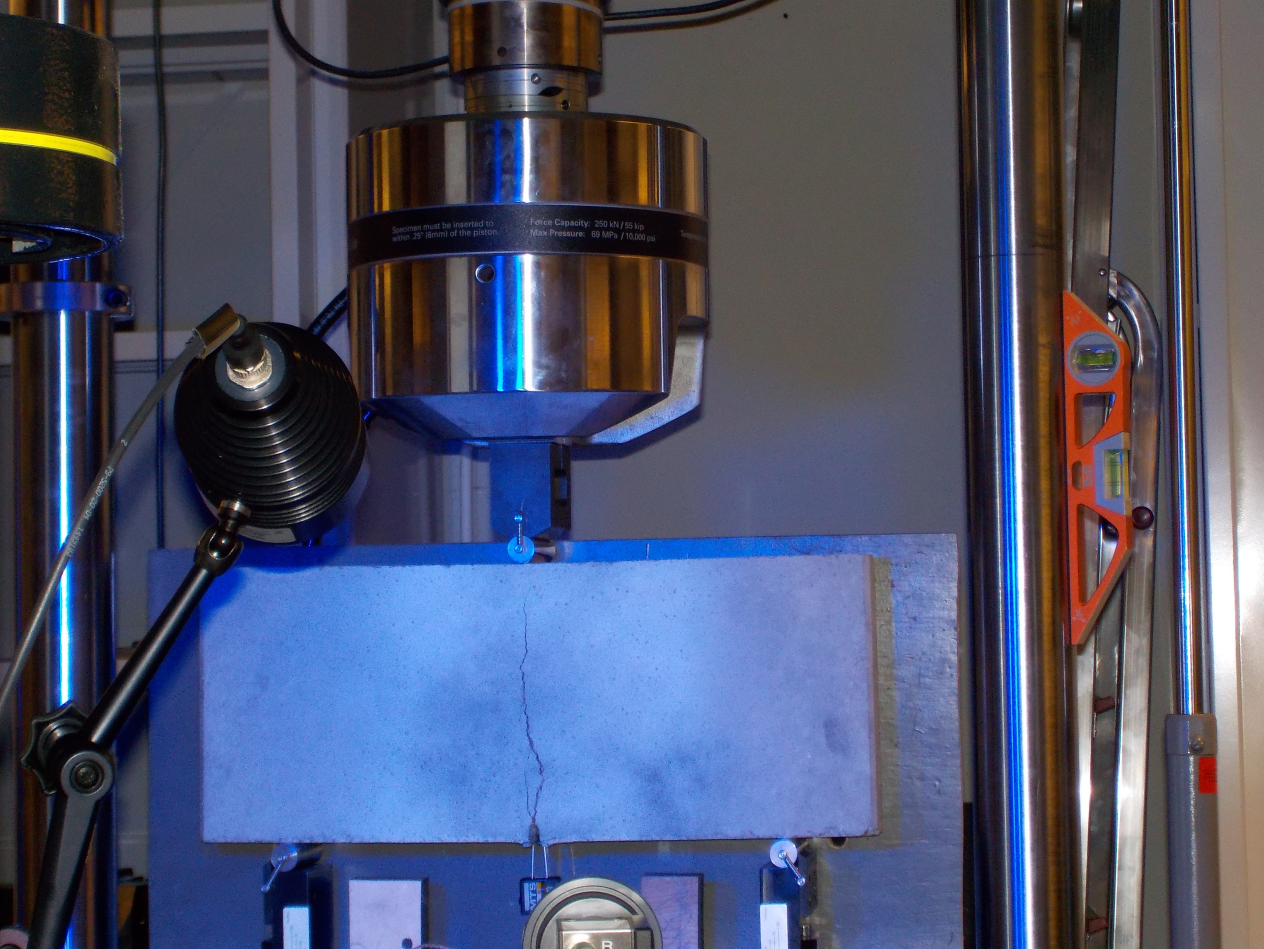
Fiber-reinforced beams during test (description in text).

Static strength tests were carried out using an MTS 810 testing machinewith a force-measuring head with a maximum value of +/− 100 kN. In addition, the extensometer number 632.02F-20 was used to measure the crack mouth opening. Static tests were carried out under conditions of monotonic three-point bending with displacement control, with the head travel speed δ = 0.1 mm/min and the use of supports located at a distance of 45 [cm] from each other, as shown in Figure 4. Static tests were carried out until complete damage of the samples was attained, as shown in Figure 5.

### 2.4. Numerical Analysis

**Figure 6:**
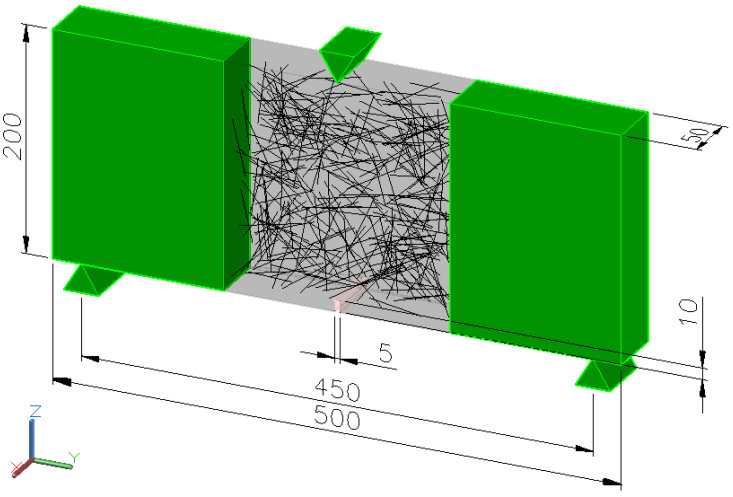
Dimensions of the sample.

**Figure 7:**
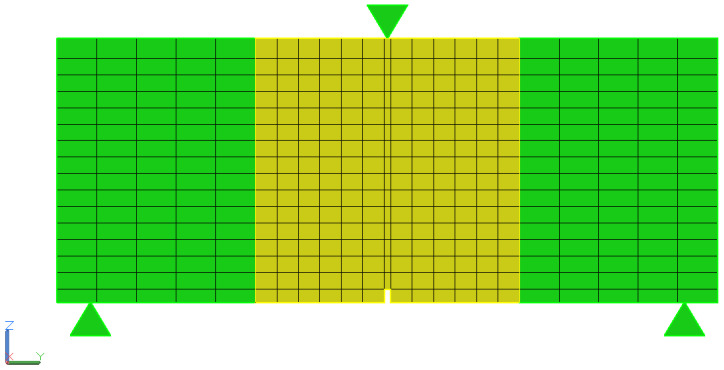
Mesh discretization.

Numerical analysis was carried out using a 3D model of beams with fibers distributed randomly. For the analysis, a uniform distribution was assumed for the position of the fiber center point and rotation relative to each axis. In order to reduce the computational time, the sample was divided into three areas: the two outer areas made of concrete modeled as isotropic and linear-elastic without fibers and the inner area made of concrete modeled using the continuous damage model and fibers, as shown in Figure 6. The size of the finite element mesh was assumed to be approx. 15 mm except for the layer near the notch, where the size along the sample was assumed as the notch width, as shown in Figure 7. Finite elements were adopted as tetrahedrons with quadratic shape functions. For analysis purposes, the constitutive model of Menétrey–Willam [32] concrete was adopted based on a preliminary test that showed that another concrete material can give solutions inconsistent with the experiment.

#### Brief Description of the Methods Used in the Analysis

Steel fibers were modeled as linear rod elements (3D 3-node beam element with quadratic shape function). In order to reflect the actual behavior of the corrugated fibers, a linear-elastic material model and a material damage model with a material property degradation method were adopted for the analysis, in which the stiffness of the element was reduced as a function of the failure parameter. In terms of the analyzed displacements (crack mouth opening up to 4 mm), such a model is consistent with the realistic behavior of fibers when pulled out of concrete [24], and there is no need to introduce an additional formulation of the interface between fibers and concrete except a simple stiff link. The fiber parameters used in the analysis are presented in Table 3 and Table 5.

A procedure written in GNU OCTAVE and LISP, similar to the method described in the publication [43], was used for random fiber generation. The number of fibers was determined based on the fiber content in the amount of $25\;\mathrm{kg}/m^{3}$ in a volume of 500 × 200 × 50 mm.

The equation can represent the stiffness matrix of the bar element subject to failure (1) [58]:

(1) $${\left[D\right]}_{d}={\left[\begin{array}{cccccc} \frac{C_{11}}{\left(1-d_{f}\right)} & C_{12} & C_{13} & 0 & 0 & 0\\ C_{21} & \frac{C_{22}}{\left(1-d_{m}\right)} & C_{23} & 0 & 0 & 0\\ C_{31} & C_{32} & \frac{C_{33}}{\left(1-d_{m}\right)} & 0 & 0 & 0\\ 0 & 0 & 0 & \frac{C_{44}}{\left(1-d_{s}\right)} & 0 & 0\\ 0 & 0 & 0 & 0 & \frac{C_{55}}{\left(1-d_{s}\right)} & 0\\ 0 & 0 & 0 & 0 & 0 & \frac{C_{66}}{\left(1-d_{s}\right)} \end{array}\right]}^{-1}$$

where $\left[C\right]$ is a compliance matrix of the undamaged material.

The failure parameters *d* are given by the following equations [58]:

(2) $$d_{f}=\left\{\begin{array}{ccc} d_{f}^{+}, & if & \lambda_{f}^{+}>0\\ d_{f}^{-}, & if & \lambda_{f}^{-}>0 \end{array}\right.$$

(3) $$d_{m}=\left\{\begin{array}{ccc} d_{m}^{+}, & if & \lambda_{m}^{+}>0\\ d_{m}^{-}, & if & \lambda_{m}^{-}>0 \end{array}\right.$$

(4) $$d_{s}=1-\left(1-d_{f}^{+}\right)\left(1-d_{f}^{-}\right)\left(1-d_{m}^{+}\right)\left(1-d_{m}^{-}\right)$$

where $d_{f}$, $d_{m}$, and $d_{s}$ are fiber, matrix, and shear variables, and λ is a failure parameter calculated from the effective stress. The signs “+” and “−” denote tension and compression, respectively.

The constitutive Menétrey–Willam [32] model was adopted as the concrete material approach, for which the failure surface is described by the following Equation (5) [32]:

(5) $$f_{MW}=\frac{c_{2}}{c_{3}}\left[\sqrt{2}\xi +r\rho \right]+\rho^{2}-\frac{1}{c_{3}}$$

where $c_{2}$ and $c_{3}$ are the parameters in the function ${\bar{R}}_{t}$, ${\bar{R}}_{c}$, ${\bar{R}}_{b}$; parameters ξ and ρ are the Haigh–Westergaard coordinates; and r is the radius defined in the paper [32]. Subscript c,t,b in previous and later equations denotes compression, tension, and bi-axial compression, respectively. The preliminary calculations show that the tension hardening–softening parameters listed in Table 4 play the most important role in the problems considered. Uniaxial tensile strength was adopted from experimental tests. Other parameters were selected based on inverse analysis for plain concrete. The ${\bar{R}}_{t}$ functions are described by the following expression [58]:

(6) $${\bar{R}}_{i}=R_{i}\Omega_{i},\;\;\mathrm{where}\;\;i=t,c\;\mathrm{or}\;b.$$

The hardening–softening behavior represented by the yield surface evolution is defined by the functions $\Omega_{t}$ and $\Omega_{c}$. These functions depend on the compression and tension hardening variables that evolve according to the work hardening expressions [58]:

(7) $$\dot{\kappa_{i}}=\frac{\alpha_{i}}{{\bar{R}}_{i}}\sigma \cdot {\dot{\varepsilon }}^{pl},\;\;\mathrm{where}\;\;i=t,c,$$

where σ and ${\dot{\varepsilon }}^{pl}$ denote stress and plastic strain, respectively. $\alpha_{c}$ and $\alpha_{t}$ are compression and tension weight functions given by [58]:

(8) $$\alpha_{c}=1-\alpha_{t}$$

(9) $$\alpha_{t}=\left\{\begin{array}{cc} 0 & tan\alpha <-2\\ \frac{1}{1+exp(-10tan\alpha )} & -2⩽tan\alpha ⩽2\\ 1 & tan\alpha >2 \end{array}\right.$$

where

(10) $$tan\left(\alpha \right)=\sqrt{6}\frac{\xi }{\rho }$$

The yield function in tension, $\Omega_{t}$, is given by an exponential softening expression where the volumetric energy dissipated during softening is proportional to mode I of the area-specific fracture energy in tension $G_{f}$ [58]:

(11) $$\Omega_{t}=exp\left(-\frac{\kappa }{a_{t}}\right)$$

where

(12) $$a_{t}=\frac{g_{ft}}{R_{t}}$$

and

(13) $$g_{ft}=max\left(\frac{G_{ft}}{L_{i}},\frac{R_{t}^{2}}{E}\right)$$

where $L_{i}$ is the effective element length and *E* is Young’s modulus, giving the following relationship of the tension yield function for the energy dissipated during softening [58]:

(14) $$\int_{0}^{\infty }\Omega_{t}d\kappa =\frac{g_{ft}}{R_{t}}$$

A detailed description of the Menétrey–Willam model can be found in [32].

**Table 5:** Material model parameters in the damage evolution law for steel fibers.

Parameter	Value
Damage initiation criteria	maximum strain
Tensile strain limit	0.1
Damage evolution law	material properties degradation
Tensile and compressive stiffness reduction	0.95

The analysis was based on a transient structural method (integration of equations of motion, Equation (15)) in order to take into account dynamic effects on the F-CMOD curve.

(15) $$M\ddot{q}+C\dot{q}+\mathrm{Kq}=F\left(t\right)$$

where M is a mass matrix, C is a damping matrix, K is a stiffness matrix, F is a load vector, q is a nodal displacement vector, $\dot{q}$ is a nodal velocity vector, and $\ddot{q}$ is a nodal acceleration vector [59]. The load was modeled as a moving punch (similar to the experiment) represented by a linear function. The self-weight of the samples was taken into account in the calculations. The numerical model was solved in Ansys software 2022 R2 [60] using the Newmark method. The analysis used the amplitude decay factor γ=0.1. The parameters of the Newmark method were determined based on the γ parameter [60]:

(16) $$\begin{array}{c} \delta =\frac{1}{2}+\gamma \\ \alpha =\frac{1}{4}{\left(1+\gamma \right)}^{2} \end{array}$$

Integrating the motion equations by means of the explicit method leads to several problems in the analyzed subject matter that make it impossible to apply. Among them, one can indicate the length of the time step necessary for a stable solution process, which leads to a very time-consuming calculation.

## 3. Results

### 3.1. Experimental Results

**Figure 8:**
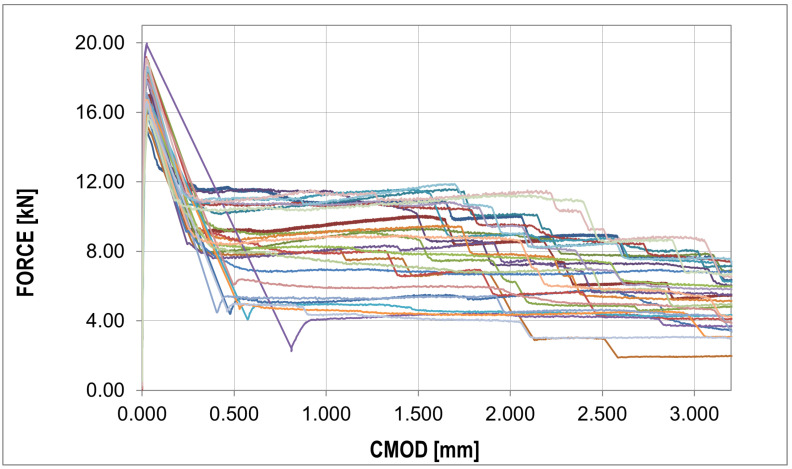
Collection of different experimental force–CMOD curves: raw data (different color different experiment, detailed description in text).

**Figure 9:**
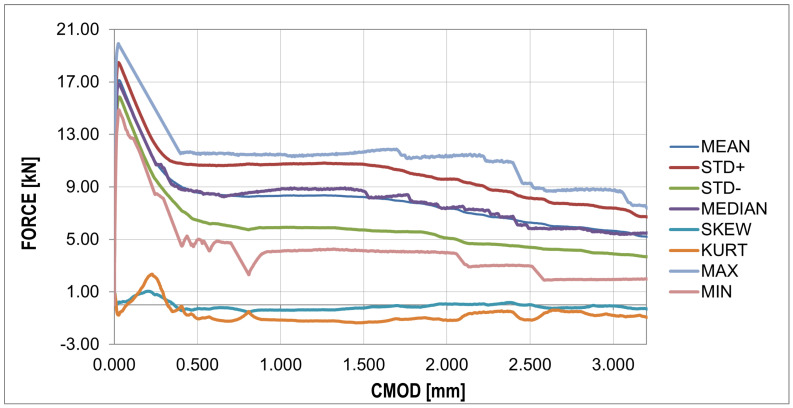
Experimentalforce–CMOD curves: statistical analysis (detailed description in text).

The F-CMOD curves represent the main results of the experimental program where some points of interest can be distinguished (Figure 8 and Figure 9). The first such point reflects a peak on the graph (maximum value) defining the tensile strength of the concrete in bending ($f_{R,B}$ in Table 4). Statistical analysis based on two statistical tests with a confidence level of 0.05 ($\chi^{2}$ with 5 degrees of freedom (X < 9.488) and the Shapiro–Wilk test (0.923 < X < 0.985)) showed perfect agreement between the probability distribution and the normal distribution, as shown in Table 6. A similar conclusion can be formulated for the graph before the bending tensile strength of concrete is exceeded. After passing the first point of interest, there is a sudden jump in the graph. A decrease in the force value is accompanied by a sudden increase in the crack mouth opening displacement (CMOD). This is the critical point of the analysis for which it was impossible to obtain sufficiently good agreement between the numerical results and experimental data. A significant amount of energy is released during monotonic loading, which, combined with the low fiber content, results in a large jump in force F and CMOD values. The sudden drop is immediately followed by non-linear hardening, after which the graph becomes almost horizontal (no increase in force or a slight increase or decrease depending on the sample). To remove statistical parameters of post-cracking strength, two points were selected: $f_{R,0.5}$ and $f_{R,1.5}$ (Table 4) at 0.5 and 1.5 crack mouth opening displacement (CMOD) values. The post-cracking deformation modulus $E_{0.5-1.5}$ was also determined (Table 4). The subsequent point of interest represents a sudden force drop in the nominal fibers’ work phase. It is associated with the strength exceeding the fibers located furthest with respect to the neutral axis, which in practice means slippage of the fiber in contact with the concrete.

For each sample, a similar failure propagation pattern can be observed after the first jump of the F-CMOD plot in the nominal phase of fiber work. After a sudden drop in force, the chart stabilizes, its shape is close to the horizontal position, and further jumps occur at intervals of about 5 mm until complete damage is attained. The jump value is approx. 2 kN.

The jumps mentioned above in the force values do not appear in the F-CMOD mean and standard deviation plots, as shown in Figure 9.

One particular point can be observed in this diagram, which also occurs for raw data Figure 8, i.e., the peak of the diagram determining the tensile strength of concrete. The median plot does not show significant deviations from the mean value, which proves the symmetry of the distribution in the CMOD function. This is confirmed by the skewness plot located around the zero values. The kurtosis plot shows mainly negative values, which proves that the intensity of the extreme values is lower than in the case of a normal distribution.

**Figure 10:**
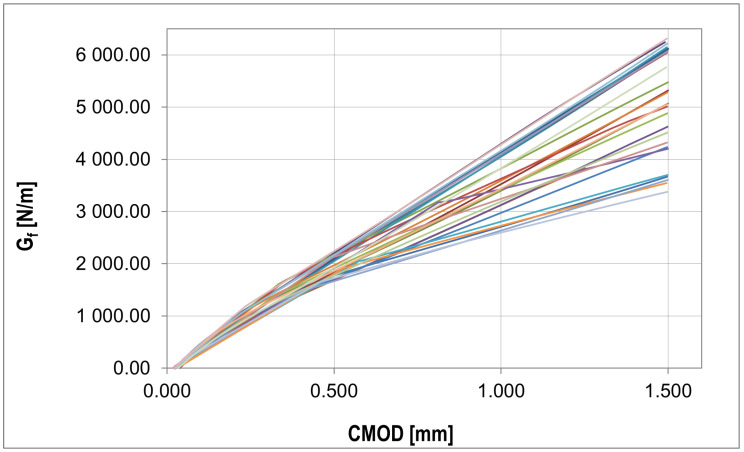
Collection of fracture energy curves for each experiment (detailed description in text).

The fracture energy presented in Figure 10 was calculated using the equation $G_{f}=\frac{W_{t}}{(d-a_{0})b}$[61], where $W_{t}$ is total energy, *d* is beam depth, $a_{0}$ is initial notch length, and *b* is beam width.

**Figure 11:**
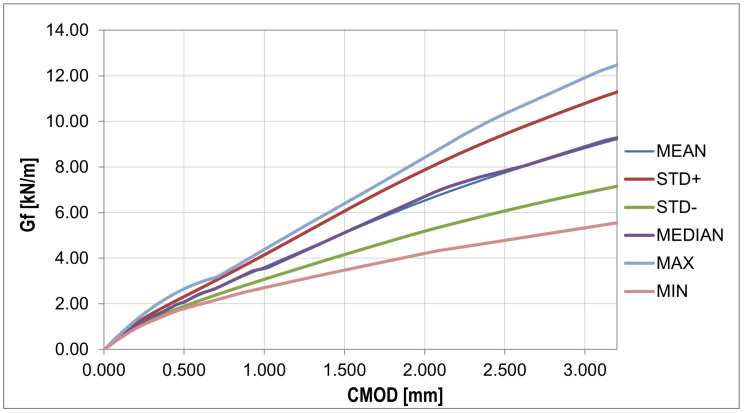
Fracture energy curves: statistical analysis (detailed description in text).

In order to analyze the random changes in the fracture energy and parameters affecting the strength and deformation process of the bent samples (Figure 11), the essential statistical characteristics summarized in Table 6 were determined. Additionally, two statistical tests were performed with the hypothesis of a normal distribution and a confidence level of 0.05: $\chi^{2}$ with 5 degrees of freedom (X < 9.488) and the Shapiro–Wilk test (0.923 < X < 0.985).

### 3.2. Numerical Results

**Figure 12:**
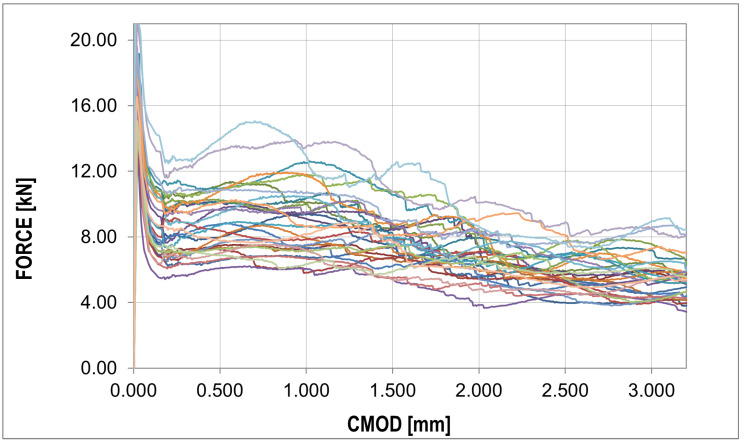
All numerical force–CMOD curves: raw data (different color different experiment, description in text).

The results of the numerical calculations are presented in Figure 12. Contrary to the experimentally captured curves, no waveforms were obtained with distinctive points in the working phase of the fibers. The graphs (Figure 12) also show local hardening and softening depending on the sample and working phase. The diagram representing the phase of concrete work before cracking shows a perfect convergence with the experimental results. This phase is dominant in the M-W model, whose compatibility with the experiment has been repeatedly confirmed.

**Figure 13:**
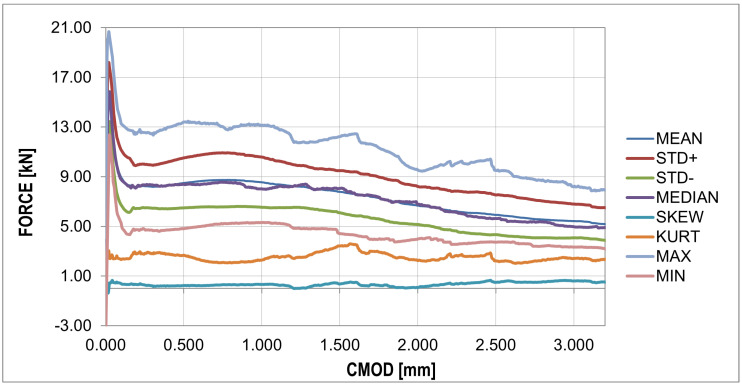
Numerical force–CMOD curves: statistical analysis.

**Figure 14:**
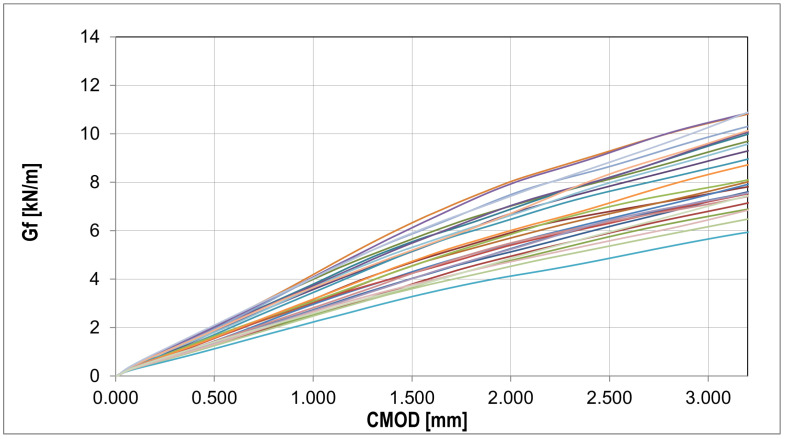
All fracture energy curves: numerical results (different color different experiment, description in text).

**Figure 15:**
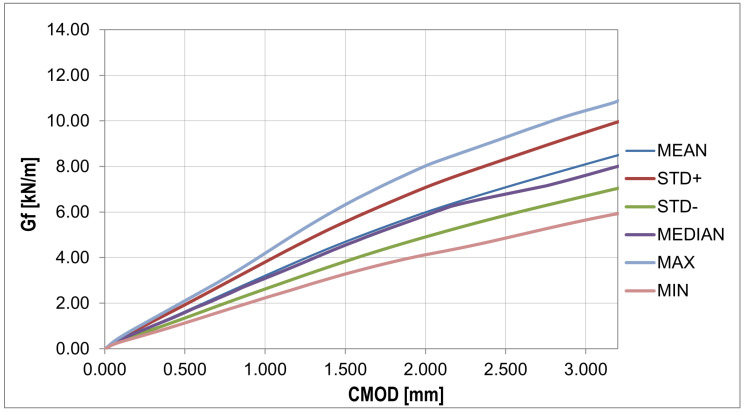
Fracture energy curves: statistical analysis.

The plots of the mean value, standard deviation, and skewness (Figure 13) give a similar pattern to that obtained from the experiment. The most significant discrepancy in the diagram was obtained for the phase after reaching the concrete tensile strength. In contrast to the plot from the experiment, the kurtosis plot shows mainly positive values. The fracture energy plots (Figure 14 and Figure 15) show a similar pattern to that obtained from the experiment, except that the curves obtained have a lower slope.

## 4. Discussion and Conclusions

### 4.1. Discussion

**Figure 16:**
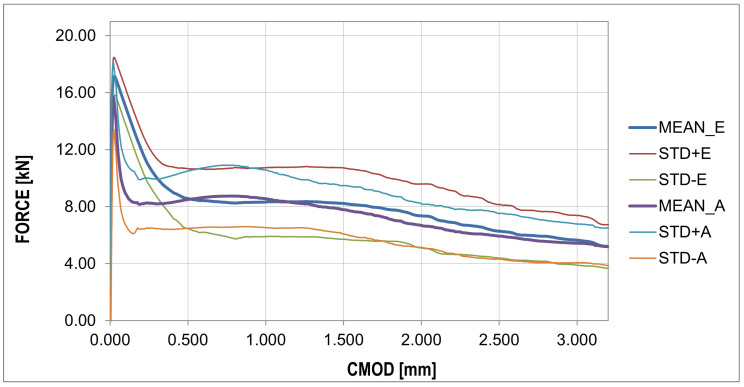
Force–CMOD curves: comparison of statistical results between numerical and experimental analysis.

**Figure 17:**
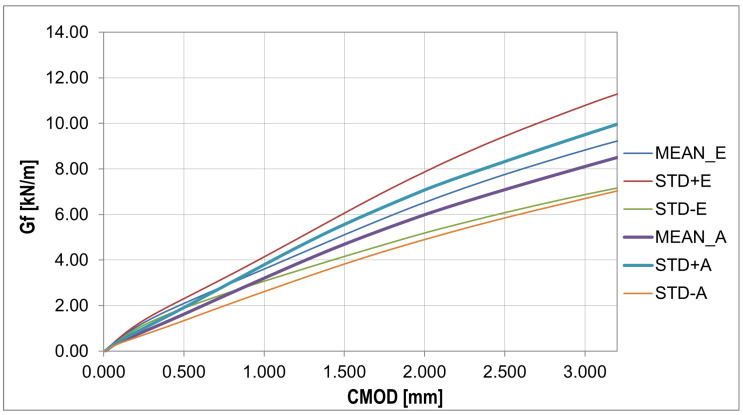
Fracture energy curves—comparison of statistical results between numerical and experimental analysis.

The analysis in this paper described sample cracking during bending with a thickness similar to the correlation length usually assumed in the stochastic concrete analysis. A thickness of 1/3 of the standard thickness of concrete samples (50 mm) with steel fibers in the amount of 25 $(\mathrm{kg}/m^{3})$ was used for the analysis. Experimental studies of three-point bending tests showed typical courses of F-CMOD with a strongly exposed elastic and non-elastic range of concrete work, as well as the phase of concrete cracking, as shown in Figure 9. In the numerical studies, obtaining a sudden decrease in strength with a simultaneous increase in CMOD was impossible. This phase of the sample work reveals the moment of transferring the accumulated energy in the process of stretching from concrete to steel fibers. The samples of standard thickness do not show such significant increases in the CMOD value. Comparing the F-CMOD waveforms obtained from the experiment (Figure 8) with the graphs from the numerical analysis (Figure 12), it can be seen that the latter have more disordered waveforms, but still show the same characteristics. However, the comparison of the waveforms obtained from the statistical analysis shows good agreement between the analyses (Figure 16 and Figure 17), outside the range of the greatest energy dissipation.

### 4.2. Conclusions

Taking into account the above discussion, the following conclusions can be made:

1. Using cracking energy parameters, the authors presented another approach to identifying the mechanical properties of a concrete composite reinforced with steel fibers.

2. The applied probabilistic analysis allowed us to obtain results describing the most important parameters of the tested material, such as peak strength, fracture toughness, crack opening displacement (CMOD), fracture energy, and modulus of elasticity after a fracture.

3. The constitutive Menétrey–Willam model was used for the analysis, which helped solve the problem.

4. To solve the problem, experimental studies were carried out on more samples (30 pcs.). The authors used a non-standard sample thickness of 1/3 of the width of standard models and an original approach to numerical calculations.

5. In experimental studies, there was a tendency for a sudden decrease in strength with a concomitant increase in CMOD. This phenomenon did not occur when testing samples with greater thickness.

6. The authors found good agreement between the experimental results and those obtained in the numerical simulation. However, the simulations did not show such significant drops in force with a simultaneous increase in CMOD.

## Figures and Tables

**Table 1:** Basic physical and chemical properties of the cement [55].

Cement Type	Setting Time Start/End		Compr. Strength	Specific Surface Area (Blaine)	Specific Gravity	${\mathrm{SO}}_{3}$	$C^{-}$	${\mathrm{Na}}_{2}O_{\mathrm{eq}}$
	(min)	(min)	(MPa)	(cm^2^/g)	(g/cm^3^)	(%)	(%)	(%)
CEM I 42.5R	176	231	57.9	3538	3.1	2.52	0.063	0.6

**Table 2:** Test results of concrete mechanical properties.

Test	ID of Mixture		
	F1	F2	ave. F1 and F2
Flow (mm)	360	350	class F2
Compressive strength 28 days (MPa)	62.46 [1.58]	62.4 [1.25]	62.43 [1.36]
Compressive strength 134 days (MPa)	51.98 [0.82]	52.42 [1.50]	52.2 [1.22]
Flexural strength 134 days (MPa)	5.58 [0.14]	5.73 [0.08]	5.66 [0.13]

**Table 3:** Steel fibers and concrete material model parameters.

Parameter	Value
Concrete density	2500 $\mathrm{kg}/m^{3}$
Concrete Young’s modulus	41.545 GPa
Concrete Poisson’s ratio	0.18
SF density	7850 $\mathrm{kg}/m^{3}$
SF Young’s modulus	4 GPa
SF Poisson’s ratio	0.3

**Table 4:** Menétrey–Willam constitutive material model parameters for concrete.

Parameter	Value
Uniaxial compressive strength $R_{c}$	62.4 MPa
Uniaxial tensile strength $R_{t}$	6.25 GPa
Biaxial compressive strength $R_{b}$	74.9 MPa
Dilatancy angle ψ	30 deg
Softening	exponential
Plastic strain at uniaxial compressive strength $\kappa_{cm}$	0.002
Plastic strain at transition form power law to exponential softening $\kappa_{cu}$	0.0035
Relative stress at start of nonlinear hardening $\Omega_{ci}$	0.3
Residual relative stress at $\Omega_{cu}$	0.75
Residual compressive relative stress $\Omega_{cr}$	0.2
Mode 1 area specific fracture energy $G_{ft}$	100 N/m
Residual tensile relative stress $\Omega_{tr}$	0.1

**Table 6:** Statistics of plain concrete and FRC.

Plain Concrete (PC)		FRC				
**Statistic**	$f_{R,B}$	**CMOD**	** $f_{R,0.5}$ **	** $f_{R,1.5}$ **	** $G_{f}$ **	** $E_{0.5-1.5}$ **
	**(MPa)**	**(mm)**	**(MPa)**	**(MPa)**	**(N/mm)**	**(MPa)**
mean	6.247	0.027	2.903	2.988	4.869	36.794
std.dev.	0.472	0.005	0.957	0.958	1.000	108.304
coeff. of var.	7.55%	17.34%	32.95%	32.08%	20.54%	294.35%
skewness	0.252	0.554	−0.464	−0.297	0.044	0.447
kurtosis	−0.699	0.085	−0.847	−1.468	−1.463	0.368
median	6.105	0.026	2.996	3.194	4.883	52.152
$\chi^{2}$(0.95,4)	6.000	3.600	5.600	6.000	2.000	6.000
Shapiro–Wilk	0.966	0.963	0.963	0.899	0.941	0.968

## Data Availability

All relevant data are contained in the present manuscript.
